# Survival-Related lncRNA Landscape Analysis Identifies *LINC01614* as an Oncogenic lncRNA in Gastric Cancer

**DOI:** 10.3389/fgene.2021.698947

**Published:** 2021-10-06

**Authors:** Huijie Wu, Jingyuan Zhou, Songda Chen, Lingyu Zhu, Mengjie Jiang, Aiqun Liu

**Affiliations:** ^1^ Department of Endoscopy Center, Guangxi Medical University Cancer Hospital, Nanning, China

**Keywords:** long noncoding RNA, gastric cancer, signature, oncogene, LINC01614

## Abstract

**Background:** Long non-coding RNAs (lncRNAs) reportedly play important roles in biomarker and tumorigenesis of gastric cancer (GC). This study aimed to determine the potential application of prognostic lncRNA signature and identified the role of *LINC01614* in carcinogenesis in GC.

**Material and Methods:** Data accessed from the Cancer Genome Atlas database was used to construct a lncRNA signature. Joint effect analysis of the signature and clinical parameters was performed to verify the clinical value of the signature. Co-expression analysis was conducted for prognostic lncRNAs and protein-coding genes. Moreover, the relative expression of *LINC01614* was validated in GC tissues and cell lines. *In vitro* and *in vivo* experiments were conducted to analyze the biological functions of the newly identified gene in GC cells.

**Results:** A seven-lncRNA (*LINC01614*, *LINC01537*, *LINC01210*, *OVAAL*, *LINC01446*, *CYMP-AS1*, and *SCAT8*) signature was identified as a promising prognostic signature in GC. Results indicated that the seven-lncRNA was involved in tumorigenesis and progression pathways. *LINC01614* expression was identified and found to be upregulated in GC tissues and cells. The study findings revealed that *LINC01614* promoted cell proliferation, migration, invasion, and epithelial-mesenchymal transition. Knockdown of *LINC01614* arrested cell cycle distribution at the G2/M phase. Further, *LINC01614* also promoted tumor growth *in vivo.*

**Conclusion:** We developed an independent seven-lncRNA biomarker for prognostic prediction and identified *LINC01614* as an oncogenic lncRNA in GC.

## Introduction

Gastric cancer (GC) is prevalent worldwide (Siegel et al., 2020). Among the Chinese population, GC is one of the most commonly reported cancers and poses a considerable public health burden (Chen et al., 2018). Despite the remarkable developments in diagnosis and treatment of GC, such as chemotherapy, radiotherapy, and surgical techniques, the overall survival has remained unsatisfactory (Yang et al., 2018). Malignant transformation and tumor progression in GC patients is diverse owing to the heterologous nature of the tumor. Moreover, the lack of availability of reliable diagnostic methods in the early stage leads to poor overall survival (OS) in GC patients (Van Cutsem et al., 2016). Recently, utilization of precision medicine has necessitated disease characterization by molecular biology approaches; the same plays a vital in GC. Replacement of the suboptimal methods with more refined and advanced methods may benefit the patients. All these objective factors contribute to the emerging demand for reliable prognosis biomarkers for GC.

The transcriptome plays an important role in disease progression and is indispensable in the investigation of carcinogenesis and pathophysiology of GC. With the development of next-generation sequencing, long non-coding RNAs (lncRNAs) have received considerable attention in the recent years. After being initially misunderstood and overlooked as “junk” in the transcriptome, lncRNAs have recently been proven to be a significant contributor in tumorigenesis. The lncRNA transcription has been reported to partly provide the signals necessary for malignant transformation (Huarte, 2015). Furthermore, lncRNAs are capable of establishing interactions with the DNA, protein, and RNA to perform regulation in multiple cancer phenotypes (Schmitt and Chang, 2016).

Utilizing the well-established methodology of genome-wide screening analysis, numerous studies have shown that the aberrant expression of lncRNA is associated with the development of various cancer types, including GC, and lncRNAs may serve as promising biomarkers in GC.(Yang et al., 2016; Zhuo et al., 2019) In this regard, systematic research is warranted to identify the function of lncRNA and to construct a promising signature based on the differential expression level of lncRNA. This study aimed to identify a promising lncRNA signature that could be associated with the OS of patients and to obtain a key lncRNA in GC.

## Materials and Methods

### RNA Sequencing Data and Annotation

The Cancer Genome Atlas (TCGA) database was accessed to extract data on 407 GC RNA sequencing (RNA-Seq) profiles. Ensemble Genomes annotation was used to define the lncRNA.

### Clinical Information

Clinical data on 380 patients corresponding to the 407 RNA-Seq profiles were obtained from TCGA. Among the data acquired, data on 368 GC patients were included in the survival analysis; data on 12 patients were excluded owing to a lack of complete survival information. Patients who met the following criteria were included in the study: 1) diagnosed GC, 2) complete RNA-Seq data, 3) complete prognostic information. The data are publicly accessible and open in TCGA, data acquisition and application in the research complied to TCGA responsible use policy and publication guidelines. Consequently, there is no need for additional agreement by any local ethics committees.

### Expression Analysis of Differentially Expressed lncRNAs in Gastric Cancer

Based on the RNA-Seq data, data on lncRNA expression profiles were extracted according to annotation. To efficiently identify the relevant biomarkers in GC, lncRNA data with a mean expression value of less than one was first excluded from the subsequent steps of screening. The edgeR package in the R language was used to screen out the differentially expressed lncRNAs (DELs) (Robinson et al., 2010). The threshold of |log2 fold change (log2FC)|> 1 as well as false discovery rate (FDR) < 0.05 were considered to screen the significant DELs.

### Construction and Analysis of the DEL Expression-Based Prognostic Signature

Association between DEL and OS of patients with GC was performed by using a univariate Cox regression model in the “Survival” package. Considering *p*-value < 0.01, DELs were regarded as statistically significant and considered to be potential candidates of the prognostic DELs. Subsequently, multivariate Cox regression and the *“step”* function which could choose a formula-based model by Akaike Information Criteria in a stepwise algorithm method in R language were used to assess the optimal combination and to identify the best model of prognostic DEL signature. The prognostic signature model was established based on a linear combination of the prognostic DEL expression levels and the multivariate Cox model. The weight was set according to the coefficient (β) that was used to multiply the regression model values calculated and output. The risk score formula was calculated as shown in the index: The Prognosis Risk Score Index = (β_1_* expression level of DEL_1_) + (β_2_* expression level of DEL_2_) +…+ (β_n_* expression level of DEL_n_). Risk score for each GC patient is calculated based on the index. Median value of the risk score for all patients was set as a cut-off point, which was then used to categorize the patients into high- and low-risk groups. Time-dependent receiver operating characteristic (ROC) curves were generated within one, three, and 5 years. The Kaplan-Meier survival curves for the cases predicted to be high-risk or low-risk were generated. Cox regression analysis and stratified analysis were further performed to demonstrate whether the signature was independent for other clinical features. Moreover, joint effect analysis was applied to explore the combined role of DEL expression-based signature and the clinical factors in prognosis.

### Prognostic DELs for Prediction and Enriched Pathway Analysis

Based on the guilt-by-association theory, the Pearson correlation coefficient was used to examine the correlation between prognostic lncRNAs and protein-coding genes (PCGs) (Rinn and Chang, 2012). The PCGs with |Pearson correlation coefficient| > 0.4 and *p* < 0.05 were considered to be potential lncRNA-related PCGs and subsequently used for conducting enrichment analysis. Gene Ontology (GO) analysis comprising molecular function (MF), cellular component (CC), and biological process (BP) was performed. The Kyoto Encyclopedia of Genes and Genomes (KEGG) is a database resource that provides information on the biological function of the genes and the associated pathways. Using the “clusterProfiler” package in R, PCGs exhibiting co-expression pattern with target DELs were analyzed based on GO and KEGG (Yu et al., 2012). The enriched terms with Benjamini-Hochberg-adjusted *p* < 0.05 were considered statistically significant.

### Clinical Samples

Twenty-two paired GC tissues and adjacent normal tissues (distance to cancer >5 cm) were obtained from patients who underwent GC resection at Guangxi Medical University Cancer Hospital. All selected samples were diagnosed by pathologists and the patients were never subjected to chemoradiotherapy or other treatments before surgery. The Ethics Committee of the Guangxi Medical University Cancer Hospital approved this study. Both informed and written consent was obtained from the participants.

### Cell Culture and Transfection

Human GC cell lines (SGC-7901, HGC-27, MGC-803, and AGS) and immortalized human gastric epithelial cell line (GES-1) were purchased from the Cell Bank of the Chinese Academy of Science, Shanghai, China. All cells were incubated in the Dulbecco’s modified Eagle medium (DMEM) (GIBCO, Waltham, MA, United States) supplemented with 10% fetal bovine serum (FBS; GIBCO). Cells were incubated at 37°C with humidified 5% carbon dioxide. At 50% cell confluency, silencing small interfering RNAs (siRNAs; GenePharma, Shanghai, China) were transfected into cells by using Lipofectamine 3,000 (Invitrogen, CA, United States). The target sequences of siRNAs are listed in Supplementary Table S1. *LINC01614*-NC and *LINC01614*-overexpressing plasmids were designed and synthesized by GenePharma Co., Ltd. and packaged into lentivirus vectors. The lentivirus vector was then transfected into SGC-7901 cells; the stably transfected SGC-7901-NC and SGC-7901-*LINC01614* cells were selected using puromycin.

### Quantitative Real-Time Polymerase Chain Reaction (qRT-PCR)

Based on the instructions of the RNAiso reagent (TaKaRa Biotechnology, Dalian, China), total RNA was isolated from the tissues and cells. All samples were quantified by using Nanodrop One (Thermo Fisher Scientific Inc., MA, United States). Primers were synthesized by Tsingke (Beijing, China). Total RNA (20 ng) was subjected to reverse transcription and then used for performing real-time PCR using a qRT-PCR Kit, according to the manufacturer’s instructions (TaKaRa Biotechnology). And qTOWER 3G (Analytik jena, jena, German) was used to determine the expression level of *LINC01614*. The relative expression of the target gene was quantified using the 2^−ΔΔCt^ method; β-actin was used as the internal reference. The primer sequences are shown in Supplementary Table S1.

### Cell Proliferation Assay

Cell proliferation assay was performed using the Cell Counting Kit-8 (CCK-8; Dojindo Laboratory, Tokyo, Japan). Cells were plated in 96-well plates at a density of 1 × 10^3^ cells/well. CCK-8 was used to determine the cell proliferation ability, according to the manufacturers’ instructions. The absorbance of each well was recorded at 450 nm using a microplate reader (BioTek, VT, United States).

### Colony Formation Assay

Cells in the logarithmic phase were isolated by pancreatic enzyme digestion and then incubated in DMEM. Thereafter, the cells were added into 6-well plates at a density of 1 × 10^3^ cells/well and cultured in an incubator until visible cell colony formation. Cells were then subjected to washing steps using phosphate-buffered saline (PBS), and fixed and stained with 0.1% crystal violet and methanol. Each experiment was repeated thrice.

### Western Blotting

Cells in each group were subjected to washing steps thrice using cold PBS and underwent lysis on ice for 10 min using RIPA (Beyotime), protease inhibitors, and phosphatase inhibitors. Total protein was extracted according to manufacturer’s instructions and the Enhanced BCA Protein Assay Kit (Beyotime) was used to determine the protein concentration, according to a selected protocol. Target proteins were isolated using 10% sodium dodecyl sulfate polyacrylamide gel electrophoresis. Subsequently, the proteins were transferred onto a polyvinylidene fluoride membrane (Sigma-Aldrich, MI, United States). The membrane was incubated with primary antibodies including Vimentin rabbit polyclonal antibody (1:1,000; Cell Signaling Technology, MA, United States), E-cadherin rabbit polyclonal antibody (1:1,000; Cell Signaling Technology) and Glyceraldehyde 3-phosphate dehydrogenase (GADPH) rabbit polyclonal antibody (1:1,000; Cell Signaling Technology) at 4°C overnight. GADPH was used as the loading control. Finally, the secondary antibody, goat anti-rabbit IgG H&L (HRP) (1:1,000; Beyotime), was added to the membrane and subjected to incubation. Thereafter, the membrane was subjected to washing steps using PBS containing Tween-20 and the bands were visualized using a chemiluminescent detection system.

### Tumor Xenograft Model

Four-to six-week-old male BALB/c nude mice were purchased from Guangxi Medical University Animal Center. All animal experiments were approved by the Animal Care & Welfare Committee of Guangxi Medical University, and animals were maintained at a specific pathogen-free environment and following the National Institutes of Health Guide for the Care and Use of Laboratory Animals. Ten mice were randomly assigned into two groups, namely SGC-7901-*LINC01614* and SGC-7901-NC. For establishment of the tumor growth model, stably transfected SGC-7901 cells (1 × 10^7^) were subcutaneously injected into the right lower groin of nude mice. The nude mice were sacrificed after 25 days. The tumor volume was calculated with the formula: volume = length × width^2^/2.

### Cell Cycle Assay

Propidium iodide (PI) stain was used to determine the cell cycle distribution. Cells were harvested at 48 h post-transfection. Thereafter, the cells were subjected to washing steps and were resuspended with PBS and centrifuged at 1,200 rpm for 5 min. After aspiration of the supernatant, cells were subjected to fixation using 1 ml pre-cooled (-20°C) 75% ethanol for 3 h at 4°C under dark conditions. Cells were then centrifuged at 12,000 rpm for 5 min and subjected to washing steps using PBS. Finally, the cells were incubated with 500 μL of the PI/RNase Staining Buffer (BD Life Sciences, NJ, United States) for 15 min at room temperature under dark conditions. The Accuri C6 Plus flow cytometer (BD Life Sciences) was used for cell cycle observation.

### Wound Healing Assay

At 48 h post-transfection, 70 μL cells were seeded into each well of ibidi Culture-Inserts. Remove the Culture-Insert to create the gap and record the image until they form an optically confluent monolayer. The cell debris was washed away using PBS. Images were recorded at 0, 36 and 48 h under ×100 magnification by Olympus I×73 inverted microscope (Olympus, Japan), respectively.

### Transwell Assay

Cell invasion was assessed using 24-well transwell chambers (Corning Life Sciences, MA, United States), with the upper surface paved using 40 μL diluted Matrigel (BD Biosciences). GC cells (5 × 10^4^ cells/well) were transferred to the upper chamber and 500 μL of DMEM containing 10% FBS was added to the lower chamber. Cells were then incubated 48 h. Thereafter, the solution in the upper chamber was removed and the cells were subjected to washing steps thrice and fixation using 4% paraformaldehyde for 30 min was performed. Cells were stained with 0.1% crystal violet and the cells that passed through the membrane were counted in five randomly chosen visual fields under ×100 magnification using an inverted microscope (Olympus, Japan).

### Gene Set Enrichment Analysis (GSEA)

The data were categorized to indicate high- and low-LINC0614 (as control group) phenotypes based on the median value. Data were analyzed using “clusterProfiler” and KEGG enrichment was performed. The number of gene set permutations was set as 1,000. Nominal *p* value and normalized enrichment score were used to perform sorting of the enriched pathways.

### Statistical Analyses

Multiple testing using the Benjamini-Hochberg procedure was performed to adjust the FDR. Log-rank test and univariate Cox regression were performed to examine the significance of the prognostic signature. The Chi-square test or the Fisher’s exact test was used to compare the validation and current dataset. Clinical features with *p* < 0.1 were included in the multivariate Cox regression model and for conducting further stratified analysis. All bioinformatics statistical analyses were performed using the R software (version 3.5.3).

The statistical analyses of experiments were performed using GraphPad Prism 8.0. All data have been expressed as mean ± standard deviation or median. Differences were calculated using the Student’s *t*-test or one-way analysis of variance followed by post hoc Dunnett’s test. A *p*-value < 0.05 was considered statistically significant.

## Results

### Differential Expression of lncRNAs in Gastric Cancer

**FIGURE 1:**
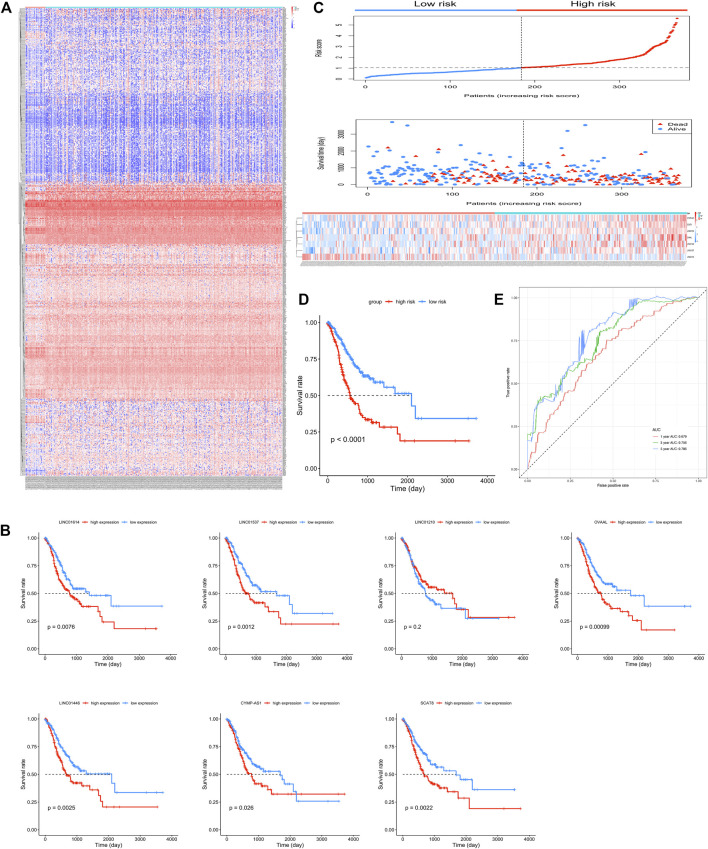
Seven candidate long non-coding RNAs (lncRNAs) of gastric cancer (GC). **(A)** Heatmap of the 858 differentially expressed lncRNAs in GC patients. **(B)** Kaplan-Meier survival curves of overall survival (OS) based on *LINC01614*, *LINC01537*, *LINC01210*, *OVAAL*, *LINC01446*, *CYMP-AS1*, and *SCAT8*, respectively, in GC patients. **(C)** Risk score and patients’ survival status distribution and expression illustrated using a heatmap of seven lncRNAs in high- and low-risk groups. **(D)** Kaplan-Meier survival curves for OS in GC patients based on the seven-lncRNA signature. **(E)** Receiver operating characteristic curve analysis of the seven-lncRNA signature for OS in GC patients.

Expression profiles of 23,151 genes obtained using RNA-Seq were downloaded from TCGA. Among these, 2,174 genes were annotated as lncRNAs and 858 lncRNAs with mean value > 1 were selected and identified as DELs. A total of 192 and 666 lncRNAs were upregulated and downregulated in GC, respectively. The heatmap of all 858 lncRNAs has been presented in Figure 1A.

### Construction of the DELs-Based Signature

Among the 858 lncRNAs, 20 with *p* < 0.01 were considered potential candidates as prognostic DELs. Subsequently, using the *“step”* function, the following seven lncRNAs were selected to construct a prognostic signature: *LINC01614*, *LINC01537*, *LINC01210*, *OVAAL*, *LINC01446*, *CYMP-AS1*, and *SCAT8*. Moreover, multivariate Cox regression was used to determine the relative contribution of potential prognostic lncRNAs toward OS prediction; additionally, our study validated that all potential DELs were independent prognostic indicators except OVAAL (Table 1). The risk score formula was imputed as per methods mentioned before. Kaplan-Meier curves showed that high expression levels of *LINC01614*, *LINC01537*, *OVAAL*, *LINC01446*, *CYMP-AS1*, and *SCAT8* were associated with an increased risk of death; *LINC01210* seemed to be the only lncRNA that followed an opposite trend and it seemed not to be a prognostic factor in GC based on log-rank test (Figure 1B). After performing calculation of the risk score for each patient, median risk score was set as a cut-off point based on which the patients were categorized into high- and low-risk groups. Survival status analysis revealed that the high-risk group was subjected to an increased risk of death. Heatmap illustration showed that the protective lncRNA, *LINC01210,* exhibited a decreased expression in the low-risk group, while others exhibited an increasing trend (Figure 1C). Patients in the high-risk group presented with a significantly decreasing median survival time of 560 days compared to those in the low-risk group (2,100 days; *p* < 0.001; Table 2 and Figure 1D). The ROC curve analysis showed an area under the curve (AUC) of 0.679, 0.756, and 0.786 for 1-, 3-, and 5-years OS prediction in GC, respectively (Figure 1E).

**TABLE 1:** The information of seven prognostic differentially expressed long non-coding RNAs which showed correlation with the overall survival of gastric cancer patients.

Ensemble ID	Gene name	*p*-value^a^	Hazard ratio^b^	Coefficient^b^	*p-*value^b^
ENSG00000230838	LINC01614	0.00250	1.1592	0.1478	0.0005
ENSG00000227467	LINC01537	0.00437	1.2209	0.1996	0.0017
ENSG00000239513	LINC01210	0.00492	0.8718	−0.1372	0.0020
ENSG00000236719	OVAAL	0.00255	1.0886	0.0849	0.0914
ENSG00000205628	LINC01446	0.00324	1.0852	0.0818	0.0111
ENSG00000235407	CYMP-AS1	0.00210	1.1348	0.1264	0.0019
ENSG00000236345	SCAT8	0.00349	1.1380	0.1293	0.0048

a Derived from the univariate Cox regression analysis

b Derived from the multivariate Cox regression analysis

**TABLE 2:** Distribution of gastric cancer patients’ clinical characteristics and subsequent analysis of prognosis.

Variable	Event/total (*n* = 368)^a^	MST (days)	HR (95% CI)	Univariate Cox *P*	Log-rank *P*
Age (years)	—	—	—	0.021	0.020
≤60	38/119	1811	1	—	—
>60	106/246	779	1.550 (1.069–2.248)	—	—
Sex	—	—	—	0.188	0.188
Female	45/133	1,043	1	—	—
Male	99/235	869	1.267 (0.890–1.803)	—	—
Lauren	—	—	—	0.44	0.439
Diffuse type	24/61	1811	1	—	—
Intestinal type	34/73	792	1.229 (0.728–2.076)	—	—
Location	—	—	—	0.478	0.478
Cardia	38/88	782	1	—	—
Noncardia	100/265	1,043	0.873 (0.601–1.269)	—	—
Tumor stage	—	—	—	<0.001	<0.001
I + II	44/159	1811	1	—	—
III + IV	90/186	675	1.943 (1.355–2.787)	—	—
Histologic grade	—	—	—	0.083	0.082
G1+G2	50/143	1,294	1	—	—
G3	90/216	801	1.359 (0.961–1.922)	—	—
Residual tumor	—	—	—	—	<0.001
R0	99/293	1,407	1	—	—
R1+R2+RX	31/49	294	3.875 (2.576–5.829)	—	—
Risk	—	—	—	<0.001	<0.001
Low	55/184	2,100	1	—	—
High	89/184	560	2.338 (1.666–3.281)	—	—

a 12 patients do not have survival information

Abbreviations: MST, median survival time; HR, hazard ratio

### Stratified Analysis and Analyses Combined With Risk Score and Clinical Parameters

**FIGURE 2:**
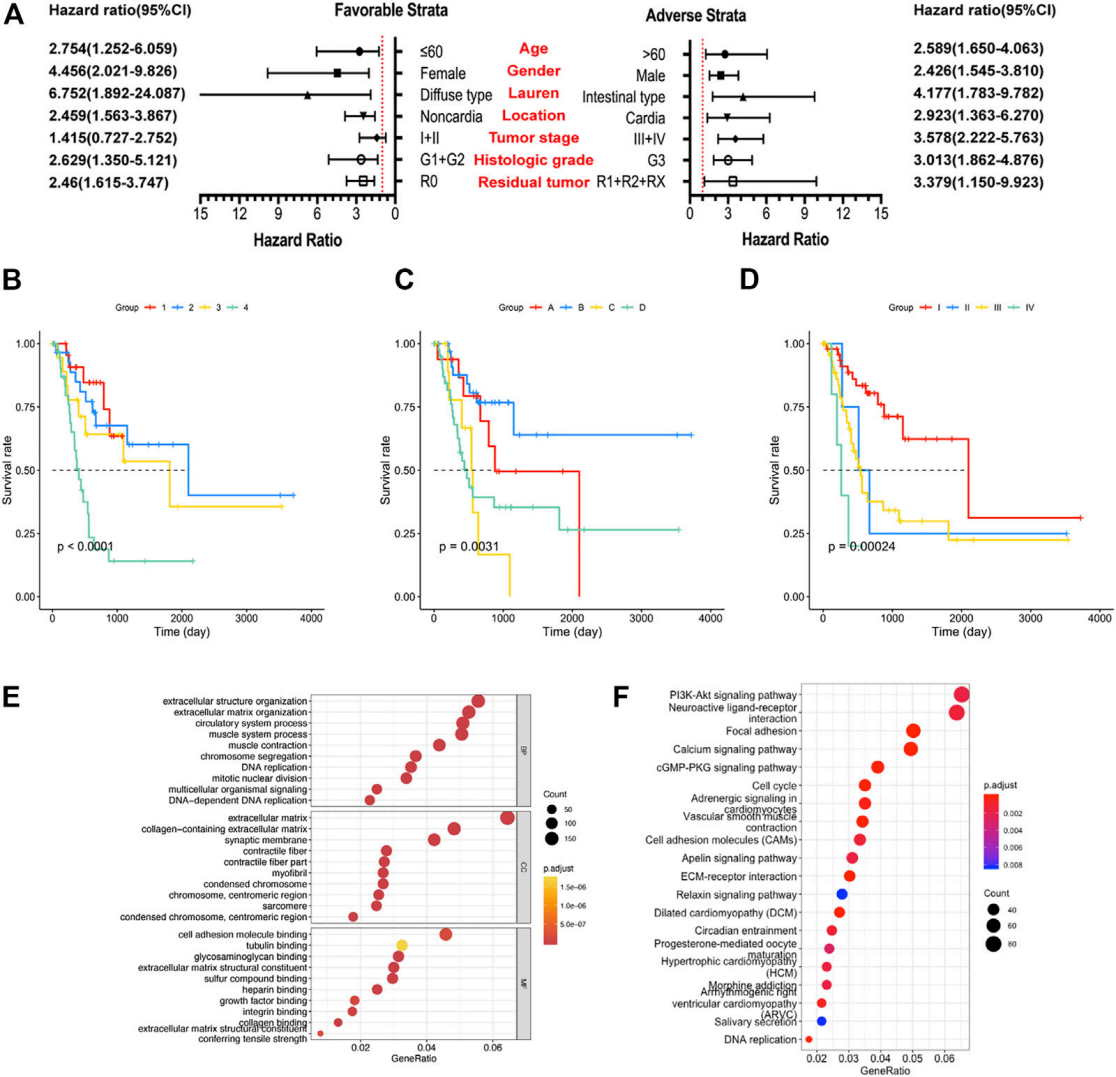
Analysis of the prognostic signature, clinical factors, and enriched pathways. **(A)** Stratified analysis of risk score and overall survival (OS) of gastric cancer (GC) patients. **(B)** Joint effect analysis of risk score and tumor stage. **(C)** Joint effect analysis of risk score and histological grade. **(D)** Joint effect analysis of risk score and residual tumor. **(E)** Gene Ontology term analysis of the biological process, cellular components, and molecular functions. **(F)** Results of the Kyoto Encyclopedia of Gene and Genome analysis.

To further confirm the combined prognostic value of clinical parameters and the prognostic signature, univariate Cox regression model was used. As shown in Table 2, risk score, age, tumor stage, and residual tumor of patients were associated with OS of GC patients. To consider all confounding factors, age, tumor stage, histological grade, and residual tumor with *p* < 0.1 were included in the multivariate Cox model and stratified analysis. After performing adjustments for the confounding factors, multivariate Cox model-based analysis showed that patients in the high-risk group were exposed to a significantly high risk of death (*p* < 0.0001, adjusted hazard ratio = 2.338; 95% confidence interval = 1.666–3.281 for OS). Stratified analysis conducted to explore the prognostic signature value under all clinical circumstances showed that the risk score could be efficiently used to predict the tumor stage, histological stage, and residual tumor in favorable and adverse strata, after subjection to adjustment for the confounding factors (Figure 2A). The joint effect analysis based on the combination of the seven-lncRNA signature and clinical features including tumor stage, histological stage, and residual tumor, indicated that the signature showed appreciable performance in predicting GC patients’ survival (Figures 2B–D; Table 3).

**TABLE 3:** Clinical factors combined with risk factors for analysis of the overall survival of patients with gastric cancer.

Group	Risk	Variable	Events/total (*n* = 368)	MST (days)	HR (95%CI)	*p* Value	Adjusted HR (95%CI)^a^	Adjusted *P*
—	—	Tumor stage	—	—	—	—	—	—
1	Low	I + II	5/23	NA	1	—	1	—
2	Low	III + IV	10/31	2,100	1.291 (0.436–3.819)	0.644	1.041 (0.335–3.237)	0.945
3	High	I + II	8/19	1811	1.909 (0.618–5.898)	0.261	2.165 (0.685–6.840)	0.188
4	High	III + IV	22/34	406	5.004 (1.879–13.328)	0.001	5.088 (1.875–13.809)	0.001
—	—	Histologic grade	—	—	—	—	—	—
A	Low	G1+G2	7/16	881	1	—	1	—
B	Low	G3	8/38	NA	0.543 (0.197–1.498)	0.238	0.575 (0.204–1.623)	0.296
C	High	G1+G2	7/13	552	2.429 (0.843–6.996)	0.100	2.833 (0.971–8.267)	0.057
D	High	G3	23/40	474	1.938 (0.829–4.534)	0.127	2.598 (1.061–6.361)	0.037
—	—	Residual tumor	—	—	—	—	—	—
I	Low	R0	12/49	2,100	1	—	1	—
II	Low	R1+R2+RX	3/5	591	2.623 (0.733–9.388)	0.138	1.729 (0.467–6.403)	0.412
III	High	R0	26/48	543	3.108 (1.561–6.188)	0.001	3.486 (1.733–7.013)	<0.001
IV	High	R1+R2+RX	4/5	262	8.239 (2.578–26.332)	<0.001	10.832 (3.229–36.34)	<0.001

a Adjusted for age, tumor stage, histologic stage and residual tumor

Abbreviations: MST, median survival time; HR, hazard ratio

### Functional Analysis and Pathway Prediction

**FIGURE 3:**
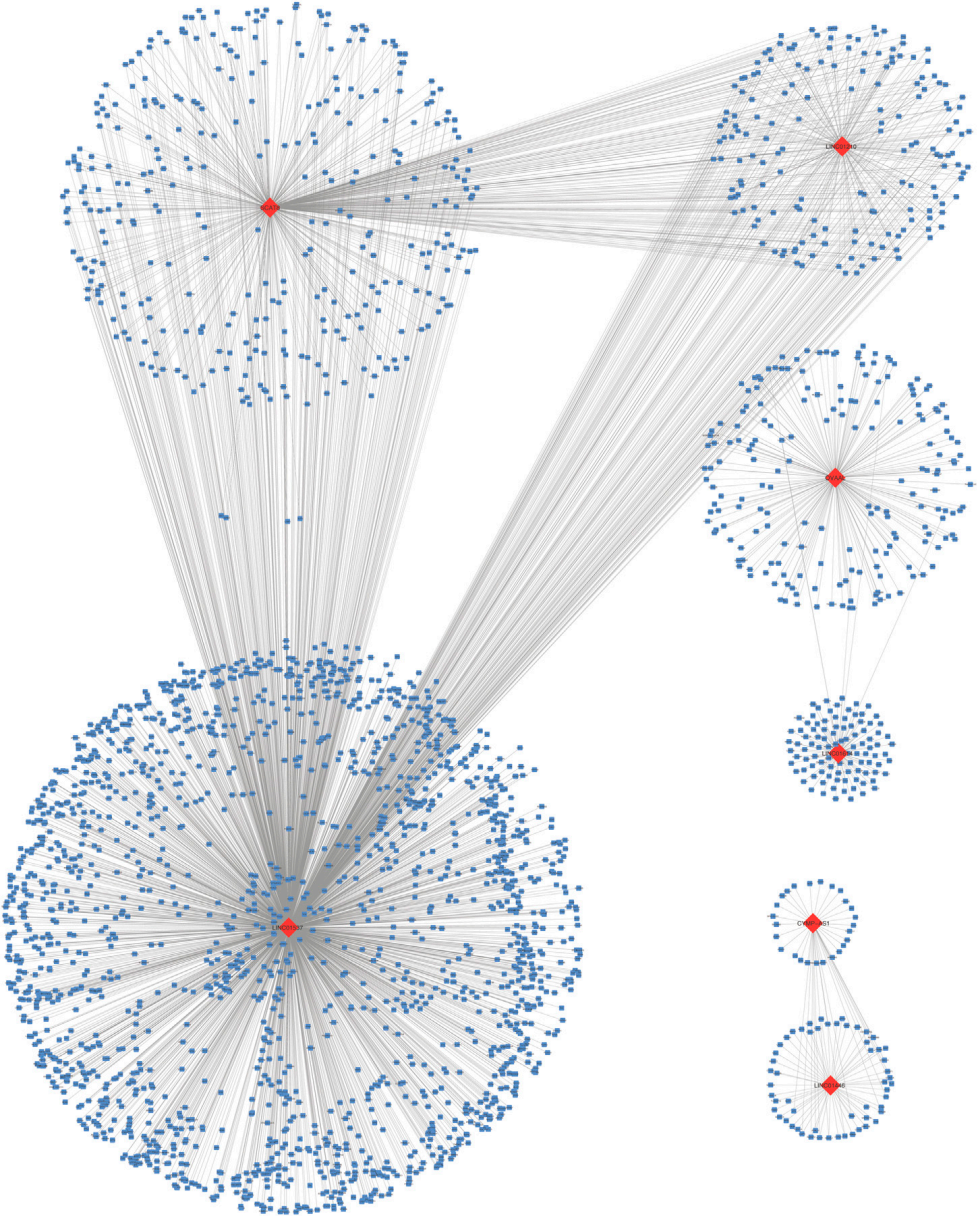
Seven long non-coding RNAs correlated protein-coding genes.

A total of 3,165 PCGs were identified as seven-lncRNA-correlated genes (Figure 3). GO enrichment using “clusterProfiler” indicated that the seven lncRNAs were significantly associated with 1065 GO terms. The top ten associated GO terms of BP, CC, and MF, including extracellular matrix and cell adhering binding, are shown in Figure 2E. KEGG enrichment confirmed that a total of 321 pathways were enriched by the target genes. The top 20 enriched pathways included phosphatidylinositol 3-kinase-protein kinase B (PI3K-Akt), focal adhesion, and cell cycle pathway (Figure 2F).

### *LINC01614* Expression Is Upregulated in Gastric Cancer

**FIGURE 4:**
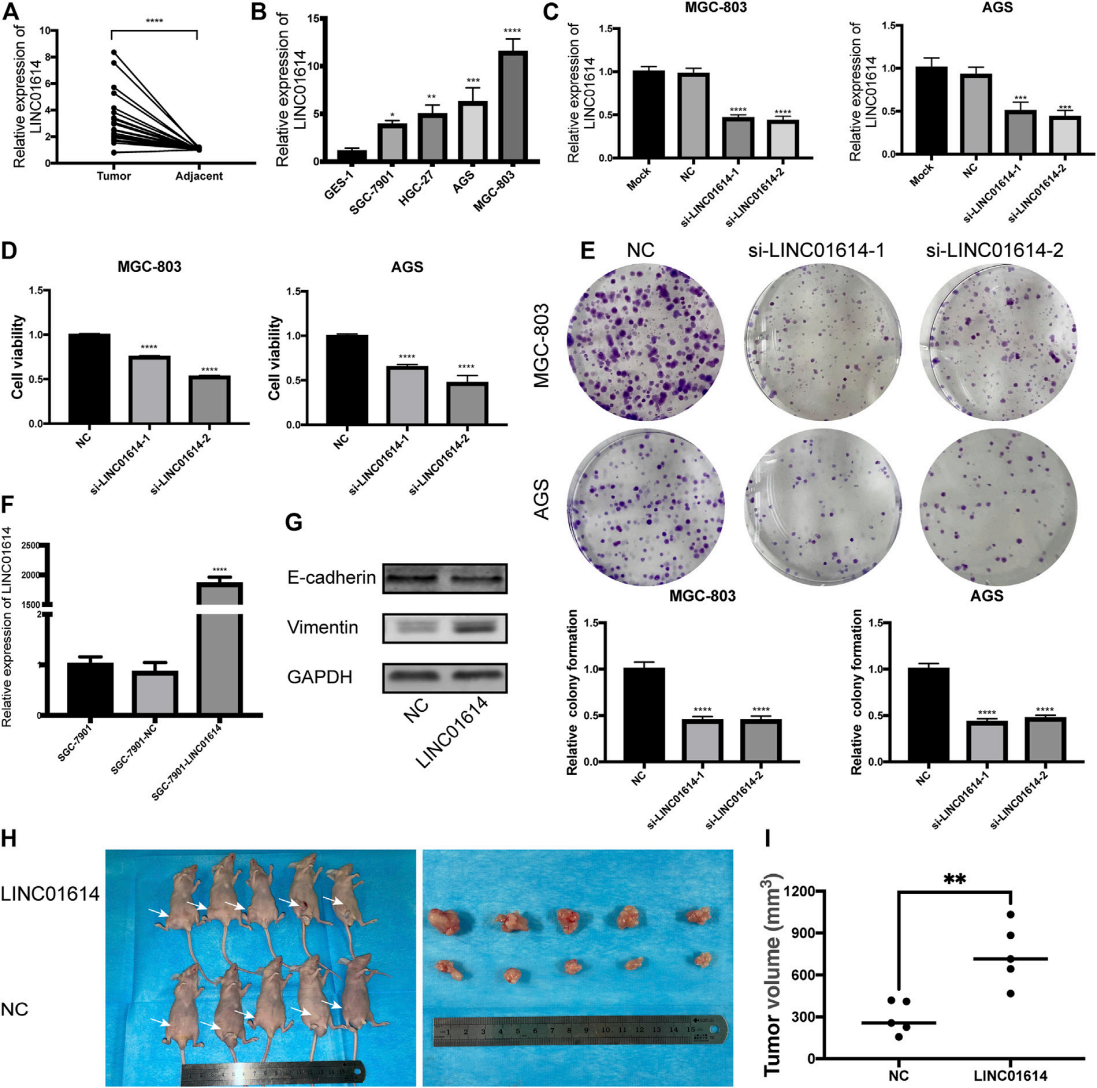
*LINC01614* expression is upregulated in gastric cancer (GC) tissues and cell lines, and promotes tumor growth **(A)** *LINC01614* expression in 22 GC tissues and paired adjacent normal tissues determined by performing quantitative real-time polymerase chain reaction (qRT-PCR). **(B)** The expression of *LINC01614* in 4 GC cell lines and immortalized human gastric epithelial cell line detected by performing qRT-PCR. **(C)** qRT-PCR analysis of the expression of *LINC01614* in MGC-803 and AGS cells after performing knockdown of *LINC01614*. **(D)** Cell Counting Kit-8 assay was used to analyze the cell proliferation of MGC-803 and AGS. **(E)** Colony formation assay was performed to detect the colony number of MGC-803 and AGS cells. **(F)** The effectiveness of *LINC01614* overexpression-based transfection was assessed through qRT-PCR. **(G)** Expression of epithelial mesenchymal transition markers was quantified using western blotting after overexpressing *LINC01614*. **(H–I)** Tumor volume between control and *LINC01614*-overexpression group showed significant difference in xenograft assay.

Similar to the findings reported by a previous study, the seven-lncRNA signature might be considered a prognostic signature in GC; *LINC01614* was the most significant lncRNA (*p* = 5 × 10^–4^). To validate the findings, *LINC01614* was selected in the following study. Through analyzing the dataset GSE95667 in GEO database, it is found that the expression of LINC01614 in gastric cancer tissues is also upregulated with the log2FC of 1.953 (*p* < 0.05). Consistent with TCGA analysis results, analysis based on qRT-PCR revealed the existence of an increased expression of *LINC01614* in the GC tissues compared to the adjacent normal tissues (Figure 4A). Consistently, *LINC01614* expression also increased in SGC-7901, HGC-27, AGS, and MGC-803 compared to GES-1 (Figure 4B, *p* < 0.05).

### *LINC01614* Promotes Proliferation *in Vitro* and *in Vivo*

AGS and MGC-803 with relatively high expression levels of *LINC01614* were used in subsequent experiments. After subjection to transfection with si-*LINC01614*-1 or si-*LINC01614*-2, expression of *LINC01614* was downregulated in MGC-803 and AGS (Figure 4C, *p* < 0.001). CCK-8 and colony formation assays revealed a decrease in the cell proliferation ability and in the colony number for cells transfected with si-*LINC01614*-1 or si-*LINC01614*-2 (Figures 4D,E, *p* < 0.01). Successful overexpression of *LINC01614* was observed in SGC-7901 cell and was validated using qRT-PCR (Figure 4F, *p* < 0.0001). Furthermore, results of western blotting showed a decrease in the protein levels of E-cadherin in the SGC-7901-*LINC01614* group, while Vimentin expression increased; *LINC01614* overexpression promoted the epithelial-mesenchymal transition (EMT) process (Figure 4G). By performing subcutaneous implantation of the transfected SGC-7901, we found that tumor size in the SGC-7901-*LINC01614* group was remarkably bigger than that observed in the SGC-7901-NC group; the observation was consistent with the outcomes of the *in vitro* experiment (Figures 4H,I).

### *LINC01614* Affects Cell Cycle Distribution and Promotes the Migration and Invasion of Gastric Cancer Cells

**FIGURE 5:**
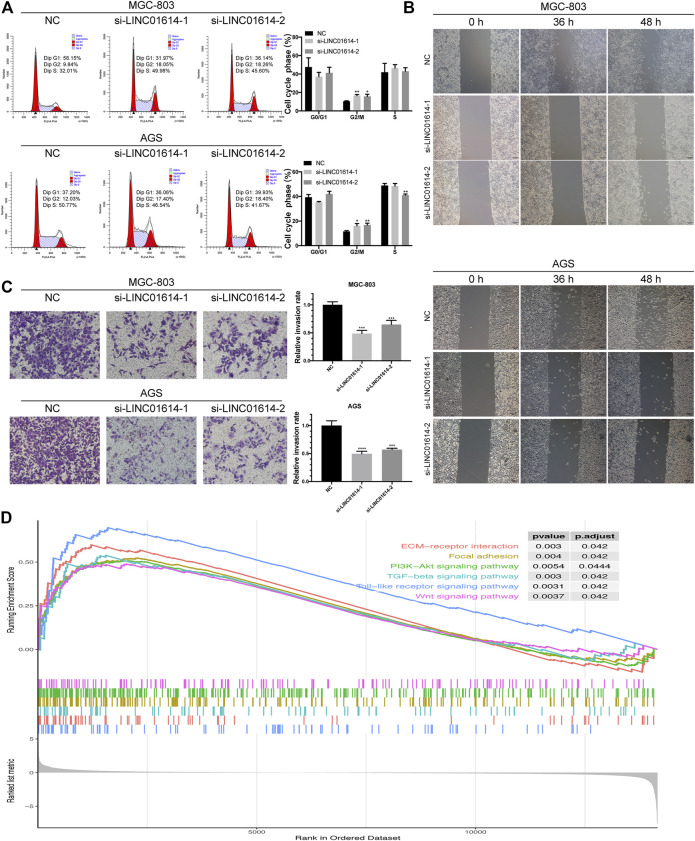
Biological effects of *LINC01614* expression exerted on cell cycle, migration, and invasion. **(A)** The cell cycle distribution of transfected MGC-803 and AGS cells was assessed by performing flow cytometry. **(B)** Wound healing assay was performed to determine MGC-803 and AGS cell migration after transfection. **(C)** The MGC-803 and AGS cell invasion after transfection was determined by performing transwell assay. **(D)** Enrichment plot generated from gene set enrichment analysis data.

Decreased expression of *LINC01614* in GC cells results in an increased percentage of cells in the G2/M phase (Figure 5A). Results indicated that the downregulated expression of *LINC01614* might lead to the arrest of cells at the G2/M phase. Wound healing assay showed that the migration ability was weakened in MGC-803 and AGS cells transfected with si-*LINC01614*-1 or si-*LINC01614*-2 compared to the NC group (Figure 5B). The number of invasive cells decreased in groups showing inhibited *LINC01614* expression compared to the NC group in transwell assay (Figure 5C). Thus, downregulated expression of *LINC01614* inhibited the migration and invasion of GC cells.

### GSEA Analysis of *LINC01614*

GSEA was used to further validate the involvement of *LINC01614* in GC pathogenesis. Enriched pathways, including PI3K-Akt signaling pathway, mitogen-activated protein kinase signaling pathway, focal adhesion, Wnt signaling pathway, Toll-like receptor signaling pathway, tumor growth factor-beta signaling pathway, and ECM-receptor interaction revealed that *LINC01614* might exert potential regulatory effect in GC through multiple mechanisms (Figure 5D).

## Discussion

TCGA, a milestone-like program, established a comprehensive dataset of mutations that occur in 33 cancer types (Weinstein et al., 2013). Based on the data on lncRNA expression profiles extracted from TCGA, the present study showed that aberrant expression levels of *LINC01614*, *LINC01537*, *LINC01210*, *OVAAL*, *LINC01446*, *CYMP-AS1*, and *SCAT8* may associated with OS in GC patients and these seven lncRNAs could be used to construct a prognostic signature in GC. The ROC analysis indicated that the signature showed appreciable performance in survival prediction within 5 years. Kaplan-Meier survival curves reiterated the same finding. And Our study showed an improvement in the survival prediction compared to the signature reported before. (Miao et al., 2017; Wang H et al., 2020).

Aberrant expression of lncRNAs and their role in GC pathogenesis have attracted the attention of multiple researchers. Xu et al. have validated that *PVT1* with upregulated expression in GC through activation of *FOXM1* transcription acts as a oncogene. (Lian et al., 2020). Additionally, a study confirmed AK023391 as a novel oncogenic lncRNA in GC that exhibited functions by mediating the PI3K-Akt signaling pathway (Huang et al., 2017). All such studies have highlighted the convincing regulatory role of lncRNAs in tumorigenesis and progression, and also lays the foundation for the conduction of future studies dedicated to unravelling the functions of lncRNA in GC. Inspiringly, some of the GC survival-related lncRNAs show commonality with the previous studies conducted on cancer. *OVAAL*, has been previously identified to play a significant role in cell survival, proliferation, and evasion from cellular senescence and contributes to cancer cell survival (Sang et al., 2018). *LINC01446* functions as an oncogene and promotes GC and glioblastoma progression and indicates a poor prognosis, an effect similar to that predicted here. (Lian et al., 2020; Zhang et al., 2018).

*LINC01614* is located on human chromosome 2q35, so far, many studies have shown that it promotes the progression of different cancers, such as lung cancer (Liu et al., 2018), breast cancer (Vishnubalaji et al., 2019), glioma (Wang Y et al., 2020) and osteosarcoma (Cai et al., 2021). Similarly, Chen et al.’ work addresses part of the same issues and reports similar results as those presented here. And the two independent pieces of work arrive at essentially the same conclusions that *LINC01614* acts as an oncogene in GC and gives added weight to the finding of both studies. Chen et al. reported that *LINC01614* has the potential to segregate a subgroup of L1 patients which was defined by authors with worse survival, however, the experiment to illustrate the relative expression of *LINC01614* in GC samples and *in vivo* study was not performed. (Chen et al., 2021). And in our study, *in vitro* and *in vivo* experiments revealed the oncogenetic character of *LINC01614* which is the most significant candidate of the signature in GC. In the study, *LINC01614* expression was observed to be upregulated in GC tissue obtained compared to the adjacent normal tissue in our cohort; upregulated expression of *LINC01614* in GC tissues and cell lines were consistent with the bioinformatics analysis results as well. These findings suggest that *LINC01614* may play a role in tumorigenesis. We then examined the function of *LINC01614* in GC cells *in vitro*, the results revealed that *LINC01614* stimulated the development of gastric cancer, it promotes cell proliferation and colony formation. The effects of knockdown of *LINC01614* using siRNAs transfection were revealed as followed: the attenuation of cell proliferation, inhibition of cell migration, invasion and EMT, leading to G2/M cell cycle arrest.

Nevertheless, the limitations of this study should be acknowledged. Despite the significant upregulation of *LINC01614* characterized here in GC along with previous reports in other cancers, the molecular mechanisms still are not clearly seen, and more explorations are also necessary to detect upstream or downstream effectors of *LINC01614* in GC progression. For another, the prognostic value of the lncRNAs signature and the expression of the lncRNA after treatment should be validated in other cohorts with large sample in future. In conclusion, this study identified a seven-lncRNA signature comprising *LINC01614* and six lncRNAs to predict OS in GC patients. Moreover, *LINC01614* expression was reported to be markedly upregulated in GC, and it may promote gastric carcinogenesis.

## Data Availability

The original contributions presented in the study are included in the article/Supplementary Material, further inquiries can be directed to the corresponding author.

## References

[B1] Cai Q. Zhao X. Wang Y. Li S. Wang J. Xin Z. (2021). LINC01614 Promotes Osteosarcoma Progression via miR-520a-3p/SNX3 axis. Cell Signal. 83, 109985. 10.1016/j.cellsig.2021.109985 33753211

[B2] Chen W. Sun K. Sun K. Zheng R. Zeng H. Zhang S. (2018). Cancer Incidence and Mortality in China, 2014. Chin. J. Cancer Res. 30, 1–12. 10.21147/j.issn.1000-9604.2018.01.01 29545714 PMC5842223

[B3] Chen Y. Cheng W. Y. Shi H. Huang S. Chen H. Liu D. (2021). Classifying Gastric Cancer Using FLORA Reveals Clinically Relevant Molecular Subtypes and Highlights LINC01614 as a Biomarker for Patient Prognosis. Oncogene 40, 2898–2909. 10.1038/s41388-021-01743-3 33742127 PMC8062268

[B4] Huang Y. Zhang J. Hou L. Wang G. Liu H. Zhang R. (2017). LncRNA AK023391 Promotes Tumorigenesis and Invasion of Gastric Cancer through Activation of the PI3K/Akt Signaling Pathway. J. Exp. Clin. Cancer Res. 36, 194. 10.1186/s13046-017-0666-2 29282102 PMC5745957

[B5] Huarte M. (2015). The Emerging Role of lncRNAs in Cancer. Nat. Med. 21, 1253–1261. 10.1038/nm.3981 26540387

[B6] Lian Y. Yan C. Lian Y. Yang R. Chen Q. Ma D. (2020). Long Intergenic Non-protein-coding RNA 01446 Facilitates the Proliferation and Metastasis of Gastric Cancer Cells through Interacting with the Histone Lysine-specific Demethylase LSD1. Cell Death Dis 11, 522. 10.1038/s41419-020-2729-0 32651355 PMC7351757

[B7] Liu A. N. Qu H. J. Yu C. Y. Sun P. (2018). Knockdown of LINC01614 Inhibits Lung Adenocarcinoma Cell Progression by Up‐regulating miR‐217 and down‐regulatingFOXP1. J. Cel. Mol. Med. 22, 4034–4044. 10.1111/jcmm.13483 PMC6111824 29934982

[B8] Miao Y. Sui J. Xu S.-Y. Liang G.-Y. Pu Y.-P. Yin L.-H. (2017). Comprehensive Analysis of a Novel Four-lncRNA Signature as a Prognostic Biomarker for Human Gastric Cancer. Oncotarget 8, 75007–75024. 10.18632/oncotarget.20496 29088841 PMC5650396

[B9] Rinn J. L. Chang H. Y. (2012). Genome Regulation by Long Noncoding RNAs. Annu. Rev. Biochem. 81, 145–166. 10.1146/annurev-biochem-051410-092902 22663078 PMC3858397

[B10] Robinson M. D. McCarthy D. J. Smyth G. K. (2010). edgeR: a Bioconductor Package for Differential Expression Analysis of Digital Gene Expression Data. Bioinformatics 26, 139–140. 10.1093/bioinformatics/btp616 19910308 PMC2796818

[B11] Sang B. Zhang Y. Y. Guo S. T. Kong L. F. Cheng Q. Liu G. Z. (2018). Dual Functions for OVAAL in Initiation of RAF/MEK/ERK Prosurvival Signals and Evasion of P27-Mediated Cellular Senescence. Proc. Natl. Acad. Sci. USA 115, E11661–E11670. 10.1073/pnas.1805950115 30478051 PMC6294934

[B12] Schmitt A. M. Chang H. Y. (2016). Long Noncoding RNAs in Cancer Pathways. Cancer Cell 29, 452–463. 10.1016/j.ccell.2016.03.010 27070700 PMC4831138

[B13] Siegel R. L. Miller K. D. Jemal A. (2020). Cancer Statistics, 2020. CA A. Cancer J. Clin. 70, 7–30. 10.3322/caac.21590 31912902

[B14] Van Cutsem E. Sagaert X. Topal B. Haustermans K. Prenen H. (2016). Gastric Cancer. The Lancet 388, 2654–2664. 10.1016/S0140-6736(16)30354-3 27156933

[B15] Vishnubalaji R. Shaath H. Elkord E. Alajez N. M. (2019). Long Non-coding RNA (lncRNA) Transcriptional Landscape in Breast Cancer Identifies LINC01614 as Non-favorable Prognostic Biomarker Regulated by TGFβ and Focal Adhesion Kinase (FAK) Signaling. Cell Death Discov. 5, 109. 10.1038/s41420-019-0190-6 31263577 PMC6591245

[B16] Wang H. Wu J. Guo W. (2020). SP1-Mediated Upregulation of lncRNA LINC01614 Functions a ceRNA for miR-383 to Facilitate Glioma Progression through Regulation of ADAM12. Ott 13, 4305–4318. 10.2147/OTT.S242854 PMC7244248 32547064

[B17] Wang Y. Zhang H. Wang J. (2020). Discovery of a Novel Three-Long Non-coding RNA Signature for Predicting the Prognosis of Patients with Gastric Cancer. J. Gastrointest. Oncol. 11, 760–769. 10.21037/jgo-20-140 32953159 PMC7475326

[B18] Weinstein J. N. Collisson E. A. Collisson E. A. Mills G. B. Shaw K. R. M. Ozenberger B. A. (2013). The Cancer Genome Atlas Pan-Cancer Analysis Project. Nat. Genet. 45, 1113–1120. The Cancer Genome Atlas Research Network. 10.1038/ng.2764 24071849 PMC3919969

[B19] Yang L. Zheng R. Zheng R. Wang N. Yuan Y. Liu S. (2018). Incidence and Mortality of Stomach Cancer in China, 2014. Chin. J. Cancer Res. 30, 291–298. 10.21147/j.issn.1000-9604.2018.03.01 30046223 PMC6037587

[B20] Yang Z. Guo X. Li G. Shi Y. Li L. (2016). Long Noncoding RNAs as Potential Biomarkers in Gastric Cancer: Opportunities and Challenges. Cancer Lett. 371, 62–70. 10.1016/j.canlet.2015.11.011 26577810

[B21] Yu G. Wang L.-G. Han Y. He Q.-Y. (2012). clusterProfiler: an R Package for Comparing Biological Themes Among Gene Clusters. OMICS: A J. Integr. Biol. 16, 284–287. 10.1089/omi.2011.0118 PMC3339379 22455463

[B22] Zhang L. Wang Q. Wang F. Zhang X. Zhang L. Tang Y. (2018). LncRNA LINC01446 Promotes Glioblastoma Progression by Modulating miR-489-3p/TPT1 axis. Biochem. Biophysical Res. Commun. 503, 1484–1490. 10.1016/j.bbrc.2018.07.067 30029885

[B23] Zhuo W. Liu Y. Li S. Guo D. Sun Q. Jin J. (2019). Long Noncoding RNA GMAN, Up-Regulated in Gastric Cancer Tissues, Is Associated with Metastasis in Patients and Promotes Translation of Ephrin A1 by Competitively Binding GMAN-AS. Gastroenterology 156, 676–691. e11. 10.1053/j.gastro.2018.10.054 30445010

