# Supplementary material for: Survival-Related lncRNA Landscape Analysis Identifies LINC01614 as an Oncogenic lncRNA in Gastric Cancer
Source: Front Genet. 2021 Oct 6;12:698947. doi: 10.3389/fgene.2021.698947 (PMC8526963; doi:10.3389/fgene.2021.698947)
Supplement: Supplementary file 1 [file Table1.docx]

## Tables

**Table 1** The information of seven prognostic differentially expressed long non-coding RNAs which showed correlation with the overall survival of gastric cancer patients

| **Ensemble ID** | **Gene name** | ***P*-value^[[1]](#footnote-1)^** | **Hazard ratio****^[[2]](#footnote-2)^** | **Coefficient^2^** | ***P-*value^2^** |
| --- | --- | --- | --- | --- | --- |
| ENSG00000230838 | LINC01614 | 0.00250 | 1.1592 | 0.1478 | 0.0005 |
| ENSG00000227467 | LINC01537 | 0.00437 | 1.2209 | 0.1996 | 0.0017 |
| ENSG00000239513 | LINC01210 | 0.00492 | 0.8718 | -0.1372 | 0.0020 |
| ENSG00000236719 | OVAAL | 0.00255 | 1.0886 | 0.0849 | 0.0914 |
| ENSG00000205628 | LINC01446 | 0.00324 | 1.0852 | 0.0818 | 0.0111 |
| ENSG00000235407 | CYMP-AS1 | 0.00210 | 1.1348 | 0.1264 | 0.0019 |
| ENSG00000236345 | SCAT8 | 0.00349 | 1.1380 | 0.1293 | 0.0048 |

**Table 2** Distribution of gastric cancer patients’ clinical characteristics and subsequent analysis of prognosis

| **Variable** | **Event/total(n=368)^[[3]](#footnote-3)^** | **MST(days)** | **HR(95% CI)** | **Univariate Cox *P*** | **Log-rank *P*** |
| --- | --- | --- | --- | --- | --- |
| Age(years) |  |  |  | 0.021 | 0.020 |
| ≤60 | 38/119 | 1811 | 1 |  |  |
| >60 | 106/246 | 779 | 1.550(1.069-2.248) |  |  |
| Sex |  |  |  | 0.188 | 0.188 |
| Female | 45/133 | 1043 | 1 |  |  |
| Male | 99/235 | 869 | 1.267(0.890-1.803) |  |  |
| Lauren |  |  |  | 0.44 | 0.439 |
| diffuse type | 24/61 | 1811 | 1 |  |  |
| intestinal type | 34/73 | 792 | 1.229(0.728-2.076) |  |  |
| Location |  |  |  | 0.478 | 0.478 |
| Cardia | 38/88 | 782 | 1 |  |  |
| Noncardia | 100/265 | 1043 | 0.873(0.601-1.269) |  |  |
| Tumor stage |  |  |  | <0.001 | <0.001 |
| I+II | 44/159 | 1811 | 1 |  |  |
| III+IV | 90/186 | 675 | 1.943(1.355-2.787) |  |  |
| Histologic grade |  |  |  | 0.083 | 0.082 |
| G1+G2 | 50/143 | 1294 | 1 |  |  |
| G3 | 90/216 | 801 | 1.359(0.961-1.922) |  |  |
| Residual tumor |  |  |  |  | <0.001 |
| R0 | 99/293 | 1407 | 1 |  |  |
| R1+R2+RX | 31/49 | 294 | 3.875(2.576-5.829) |  |  |
| Risk |  |  |  | <0.001 | <0.001 |
| Low | 55/184 | 2100 | 1 |  |  |
| High | 89/184 | 560 | 2.338(1.666-3.281) |  |  |

**Table 3** Clinical factors combined with risk factors for analysis of the overall survival of patients with gastric cancer

| **Group** | **Risk** | | | **Variable** | **Events/total(n=368)** | | **MST(days)** | **HR(95%CI)** | | ***P* value** | **Adjusted HR(95%CI)****^[[4]](#footnote-4)^** | **Adjusted *P*** |
| --- | --- | --- | --- | --- | --- | --- | --- | --- | --- | --- | --- | --- |
|  | |  | Tumor stage | | |  |  | |  |  |  |  |
| **1** | | Low | I+II | | | 5/23 | NA | | 1 |  | 1 |  |
| **2** | | Low | III+IV | | | 10/31 | 2100 | | 1.291(0.436-3.819) | 0.644 | 1.041(0.335-3.237) | 0.945 |
| **3** | | High | I+II | | | 8/19 | 1811 | | 1.909(0.618-5.898) | 0.261 | 2.165(0.685-6.840) | 0.188 |
| **4** | | High | III+IV | | | 22/34 | 406 | | 5.004(1.879-13.328) | 0.001 | 5.088(1.875-13.809) | 0.001 |
|  | |  | Histologic grade | | |  |  | |  |  |  |  |
| **A** | | Low | G1+G2 | | | 7/16 | 881 | | 1 |  | 1 |  |
| **B** | | Low | G3 | | | 8/38 | NA | | 0.543(0.197-1.498) | 0.238 | 0.575(0.204-1.623) | 0.296 |
| **C** | | High | G1+G2 | | | 7/13 | 552 | | 2.429(0.843-6.996) | 0.100 | 2.833(0.971-8.267) | 0.057 |
| **D** | | High | G3 | | | 23/40 | 474 | | 1.938(0.829-4.534) | 0.127 | 2.598(1.061-6.361) | 0.037 |
|  | |  | Residual tumor | | |  |  | |  |  |  |  |
| **I** | | Low | R0 | | | 12/49 | 2100 | | 1 |  | 1 |  |
| **II** | | Low | R1+R2+RX | | | 3/5 | 591 | | 2.623(0.733-9.388) | 0.138 | 1.729(0.467-6.403) | 0.412 |
| **III** | | High | R0 | | | 26/48 | 543 | | 3.108(1.561-6.188) | 0.001 | 3.486(1.733-7.013) | <0.001 |
| **IV** | | High | R1+R2+RX | | | 4/5 | 262 | | 8.239(2.578-26.332) | <0.001 | 10.832(3.229-36.34) | <0.001 |

**Table S1** Sequences of small interfering RNAs and polymerase chain reaction primers

|  | **Sequence** |
| --- | --- |
| si-LINC01614-1 | F: 5’-GCCAUUUCCCAAAUGUCAATT-3’ |
|  | R: 5’-UUGACAUUUGGGAAAUGGCTT-3’ |
| si-LINC01614-2 | F: 5’-GCUAGAAGUGUGACUGAAATT-3’, |
|  | R: 5’-UUUCAGUCACACUUCUAGCTT-3’ |
| β-Actin | F: 5’-CTGGAACGGTGAAGGTGACA-3’ |
|  | R: 5’-CTGGAACGGTGAAGGTGACA-3’ |
| LINC01614 | F: 5’-AACCAAGAGCGAAGCCAAGA-3’ |
|  | R: 5’-GCTTGGACACAGACCCTAGC-3’ |

1. Derived from the univariate Cox regression analysis [↑](#footnote-ref-1)
2. Derived from the multivariate Cox regression analysis [↑](#footnote-ref-2)
3. 12 patients do not have survival information

   Abbreviations: MST, median survival time; HR, hazard ratio [↑](#footnote-ref-3)
4. Adjusted for age, tumor stage, histologic stage and residual tumor

   Abbreviations: MST, median survival time; HR, hazard ratio [↑](#footnote-ref-4)
